# Baseline Pneumococcal IgG Levels and Response to 23-Valent Pneumococcal Polysaccharide Vaccine among Adults from Beijing, China

**DOI:** 10.3390/vaccines11121780

**Published:** 2023-11-29

**Authors:** Shanshan Zhou, Min Lv, Shuang Bai, Weixin Chen, Wei Zhao, Jian Wang, Ao Zhang, Jing Li, Hui Xie, Yanqing Gao, Dongmei Li, Jiang Wu

**Affiliations:** 1 Beijing Center for Disease Prevention and Control, No. 16, Hepingli Middle Street, Dongcheng District, Beijing 100013, China; 2 Daxing District Center for Disease Control and Prevention of Beijing, Beijing 102600, China

**Keywords:** *Streptococcus pneumoniae*, vaccine, baseline, IgG antibody, ELIS, immunogenicity

## Abstract

Purpose: To investigate the baseline levels of serotype-specific IgG antibodies to *Streptococcus pneumoniae (S. pneumoniae)* and assess their impact on the assessment of vaccine immunogenicity. Methods: We used a subset of serum samples from a randomized controlled trial. The blood of 584 healthy participants was collected on day 0 before and day 28 after the 23-valent pneumococcal polysaccharide vaccine (PPSV23) vaccination. Serotype-specific IgG against PPSV23-covered serotypes were measured by the World Health Organization (WHO) reference enzyme-linked immunosorbent assay (ELISA). Vaccine immunogenicity was compared using conversion rates (proportion of participants with IgG levels following immunization that are 2-fold greater than the baseline) and geometric mean fold rises (GMFRs) between the two groups, which were grouped according to pre-vaccination (baseline) IgG antibody levels. Results: Our data showed that over half of individuals have baseline IgG levels for 15 out of 23 serotypes above 1.3 µg/mL, and geometric mean concentrations (GMCs) were generally higher in the elderly group and the female group; significant differences were found in 15 serotypes for vaccine immunogenicity based on the seroconversion rate or GMFRs between individuals with baseline IgG ≥ 1.3 µg/mL and individuals with baseline IgG < 1.3 µg/mL. The seroconversion rate decreased with the increase of baseline IgG levels according to a linear regression model. Conclusions: The assessment of vaccine immunogenicity could be impacted by the fact that many adults had high baseline antibody levels. This study is registered in the Chinese Clinical Trial Registry, number NCT05298800.

## 1. Introduction

*Streptococcus pneumoniae* (*S. pneumoniae*) is the most common opportunistic pathogen that colonizes the human nasopharynx and is responsible for a wide range of infections, including otitis media, community-acquired pneumonia, septicemia and meningitis. Its capsular polysaccharide is one of the most important virulence factors that can induce protective antibodies [1]. The composition of capsular polysaccharide divides *S. pneumoniae* into multiple serotypes, and more than 100 serotypes have been identified so far [2,3]. Pneumococcal disease causes over 1.6 million deaths annually worldwide, mainly in young children and the elderly, and is listed by the World Health Organization (WHO) as a kind of “very high priority” for vaccine prevention [4,5,6].

Pneumococcal polysaccharide vaccine (PPSV) and pneumococcal conjugate vaccine (PCV) are the two main types of vaccine currently used in adults, both of which have been demonstrated as effective for invasive pneumococcal disease (IPD). Although, unlike PCV, PPSV performs poorly in terms of mediating protection from carriage [7,8]; the 23-valent pneumococcal polysaccharide vaccine (PPSV23) is recommended for the elderly and at-risk adults to protect against pneumococcal diseases because of its multiple coverage and cost-effectiveness [9,10]. The total amount of anti-capsular IgG measured by ELISA and the functionality antibodies detected by opsonophagocytosis assay (OPA) are both the gold standard immunological measurements for pneumococcal vaccine licensure [8,11]. The ELISA assay was well developed as WHO-recommended third-generation ELISA (WHO-referenced ELISA) and a WHO Training Manual provides a detailed description of the detection processes (https://www.vaccine.uab.edu/uploads/mdocs/ELISAProtocol(007sp).pdf (accessed in 2000)).

A 2-fold increase in antibody concentration levels following vaccination has been linked to efficacy in clinical trials and licensure of PPSV [12], and which along with geometric mean fold rise (GMFR) are usually used in a post-marketing immunogenicity evaluation of vaccines. However, it has been demonstrated that obtaining a high-fold rise from the baseline is difficult for people with high preimmunization antibody concentrations [13,14]. Adults have naturally acquired pneumococcal antibodies to the prevalent circulating pneumococcal serotypes via repeated colonization or infection. Baseline levels are able to be influential for an accurate assessment of the vaccine immunogenicity. A large number of studies have focused on the immunogenicity of pneumococcal vaccine without taking local distribution of the baseline antibody levels into consideration [15,16,17,18]. Moreover, few studies have reported the distribution of baseline anti-pneumococcal IgG levels in all PPSV23 serotypes using the WHO ELISA due to the labor-intensive and technically challenging aspects. Specific IgG concentrations about the *S. pneumoniae* serotype in previous research measured by non-standard methods, such as multiplex bead array assays, showed variation in the assay findings and erratic correlation with ELISA performed by several laboratories [11,19]. Baseline levels reflect, to some extent, the immunity acquired by the population through healthy carriage or natural infection [20,21], and it could be helpful to optimize vaccine serotype compositions. Previous studies show that antibody levels remain higher in vaccination populations than unvaccinated populations ten years after vaccine immunization [9]; thus, it will be difficult to obtain the distribution of baseline levels once mass vaccination has been introduced. Since April 2018, Beijing has been carrying out a policy of free PPSV23 vaccinations for people over 65 years of age. Prior to providing free pneumococcal vaccination extensively in a region, it is crucial to investigate baseline polysaccharide serotype-specific antibody levels. 

Herein, we investigated the levels of *S. pneumoniae* serotype-specific IgG antibodies that are detected by the WHO ELISA in pre- and post-vaccination sera from individuals who were immunized with the PPSV23 vaccine. This paper focuses on describing the distribution of PPSV23-related serotype-specific IgG antibody baseline levels and we used a threshold of 1.3 µg/mL to classify groups of pre-vaccination IgG concentrations, which the AAAAI (American Academy of Allergy, Asthma, and Immunology/American College of Allergy, Asthma, and Immunology) defines as protective concentrations against infection and utilized it to diagnose specific antibody deficiency [22]. Moreover, we explored whether the baseline levels would affect the evaluation of vaccine immunogenicity.

## 2. Materials and Methods

### 2.1. Participants and Clinical Samples

This is a sub-study of a randomized controlled trial that evaluated the safety and efficacy of a COVID-19 inactivated vaccine in combination with a PPSV23 vaccine or a quadrivalent inactivated influenza vaccine. Healthy adults over 18 years were recruited from communities in Beijing, China, between December 2021 and March 2022. They were divided into the middle-aged group (18–59 years) and the elderly group (≥60 years). Main exclusion criteria: (1) participants with a history of pneumococcal vaccination within five years; (2) participants who had received the seasonal flu vaccine of the current year; (3) participants with a history of documented COVID-19; (4) participants who had severe allergic reactions to vaccines; (5) fever at the time of vaccination, acute exacerbation of a chronic disease, or patients with uncontrolled severe chronic disease, or suffering from an acute illness. To investigate the immunogenicity of the PPSV23 vaccine in this sub-study, serum samples were collected on day 0 before and day 28 after PPSV23 vaccination and PPSV23 co-vaccination with a COVID-19 inactivated vaccine or PPSV23 in combination with a quadrivalent inactivated influenza vaccine, and stored at −80 °C before analysis.

### 2.2. Vaccine 

Vaccines were provided by Sinovac Biotech Ltd., Beijing, China. All vaccines were administered intramuscularly. Each 0.5 mL dose of PPSV 23 (S20200027) contains 25 μg of the purified pneumococcal capsular polysaccharides for each of 1, 2, 3, 4, 5, 6B, 7F, 8, 9 N, 9 V, 10A, 11A, 12F, 14, 15B, 17F, 18C, 19A, 19F, 20, 22F, 23F, and 33F. It was licensed in China in 2020 and recommended for pneumococcal-susceptible individuals aged 2 years and older. Each 0.5 mL dose of inactivated influenza vaccine ( S20200010) was composed of 15 μg of each antigen of influenza A(H1N1) and A(H3N2), as well as two influenza B strains (Victoria and Yamagata lineages), which was licensed in China in 2020. The commercially available COVID-19 vaccine ( S20210003) contained 600SU of inactivated SARS-CoV-2 antigen.

### 2.3. S. pneumoniae Serotype-Specific IgG Detection

A vaccine-associated serotype-specific IgG antibody was detected by the WHO ELISA according to the WHO Training Manual. Briefly, the 96-well medium binding microtiter plate (Greiner Bio-One; Frickenhausen, Germany) was coated with the serotype-specific pneumococcal polysaccharide antigen to be tested (American Type Culture Collection (ATCC); Manassas, VA, USA; with the exception of pneumococcal polysaccharide serotype 3, which was obtained from Yunnan Walvax Biotech Co., Ltd. Kunming, Yunnan, China) at the optimal coating concentration (1 μg/mL for serotypes 1, 2, 5, 14, 17F, 19A, 20, and 33F; 1.5 μg/mL for serotype 22F; 2 μg/mL for serotypes 7F, 9 N, 18C; 3 μg/mL for serotype 11A; 5 μg/mL for serotype 4, 9 V, 10A, 12F, and 15B; 10 μg/mL for serotype 3, 6B, 8, 19F, and 23F), and incubated overnight. Sera were pre-diluted (1:200 for human samples; 1:1000 for quality control (QC); 1:500 for 007sp) and double preabsorbed by the absorption solution consisting of 5 μg/mL pneumococcal polysaccharide cell wall (C-PS) (Statens Serum Institute (SSI); Copenhagen, Denmark) and 5 μg/mL 22F capsular polysaccharide or 10 μg/mL Multi-C-PS (SSI, Copenhagen, Denmark) alone in antibody buffer [23]. The pre-absorbed sera, standard serum (007sp), and QC serum were diluted using a 2.5-fold serial dilution in absorption solution and incubated for 30 min. The following procedure was automated on the Tecan Freedom EVO 150 ELISA platform (Tecan Australia Pty Ltd.; Männedorf, Switzerland), and strictly adhered to the WHO ELISA protocol. In summary, the antigen-coated microtiter plates were washed five times, and 50 µL of serum dilution was added to each well and incubated for two hours. After washing the microtiter plate, 100 µL of anti-human IgG alkaline phosphatase antibody was added and the mixture was incubated for two hours. After washing the plates, substrate solution (1 mg/mL p-nitrophenyl phosphate in diethanolamine) was added, then incubated for two hours. before the addition of stop solution (50 µL 3 M NaOH). All incubation was conducted at room temperature. Plates were read at 405 nm and 620 nm. Optical density values were transformed into concentrations using a standardized curve-fitting four-parameter logistic-log equation [24]. Data inspection rules are according to page 11 of 29 in the WHO reference ELISA protocol.

### 2.4. Statistical Analysis

The results of antibody levels are reported as median concentrations and GMCs with two-sided 95% CIs. A two-tailed Mann–Whitney U test was used to analyze differences between GMCs groups and between GMFRs groups. The differences between groups in seroconversion rates were determined by Pearson chi-squared tests. A *p*-value of <0.05 was reported as statistically significant. The statistical analysis was performed using GraphPad Prism 8 and R, version 4.0.2. Pre-immunization antibody concentrations below the level of reporting (<0.1 µg/mL) were assigned 0.05 µg/mL.

## 3. Results

### 3.1. Study Population

In this study, 327 middle-aged adult participants (18–59 years) and 292 elderly adult participants (≥60 years) ranging from 18 to 87 years were recruited in Beijing, China. Among them, 309 adult participants (median age 41 years; 180 females and 129 males) and 275 elderly participants (median age 65 years; 158 females and 117 males) completed pneumococcal vaccine immunization or a combination immunization of pneumococcal vaccine and another vaccine (COVID-19 inactivated vaccine or quadrivalent inactivated influenza vaccine).

### 3.2. Distribution of Pre-Vaccination (Baseline) IgG Antibodies

**Figure 1:**
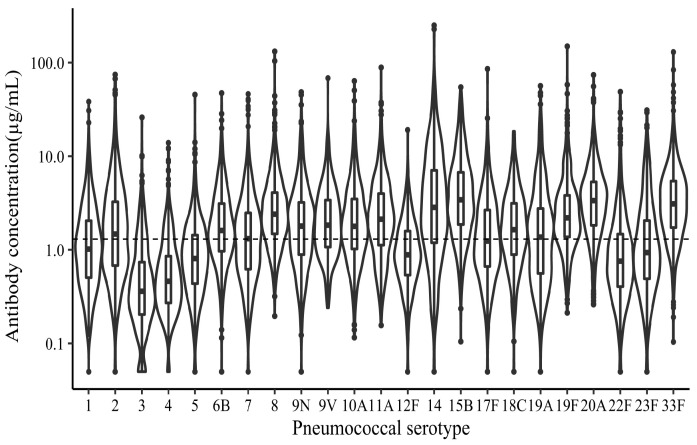
Distribution of IgG antibody concentrations of the 23 pneumococcal serotypes before vaccination. Violin plot of IgG antibody concentrations for each of PPSV23-related serotypes. Box and whisker plot of median antibody concentrations, interquartile ranges (25th and 75th percentiles), and minimum/maximum values. Antibody concentration is shown on the log scale, dashed line drawn at the value of 1.3 µg/mL.

**Figure 2:**
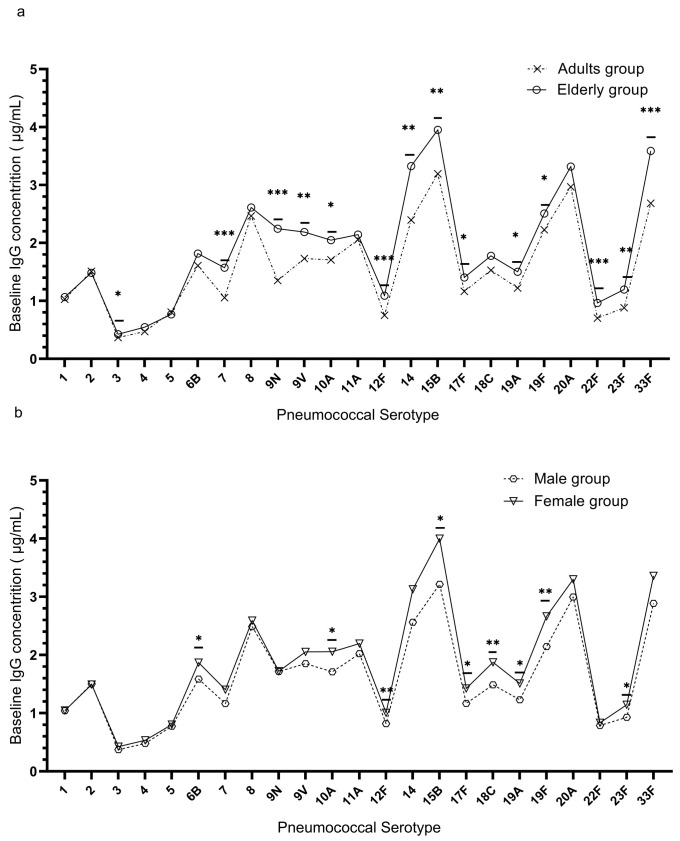
The GMCs for baseline anti-pneumococcal IgG concentrations of PPSV23-covered serotypes among age and agender groups. (**a**) The GMCs for baseline anti-pneumococcal IgG concentrations in the middle-aged and elderly groups. (**b**) The GMCs for baseline anti-pneumococcal IgG concentrations in the male and female groups. *, *p* < 0.05; **, *p* < 0.01; ***, *p* < 0.001 (Mann–Whitney U test).

The distribution of baseline concentrations for each serotype in the healthy adults over 18 years was shown in the violin plot (Figure 1). The percentage of individuals with a baseline IgG concentration ≥ 1.3 μg/mL was more than 50% in 15 out of 23 serotypes except 8 serotypes (1,3, 4, 5,12F,17F, 22F, and 23F). The geometric mean concentrations (GMCs) for baseline anti-pneumococcal IgG concentrations were generally higher in the elderly group than in the middle-aged group except for serotype 2 and serotype 5, with 14 out of 23 serotypes having statistically significant differences (3, 7F, 9N, 9V, 10A, 12F, 14, 15B, 17F, 19F, 22F, 23F, and 33F) (Figure 2a; Supplemental Table S1). The baseline IgG levels in the female group were higher compared to the male group except for serotype 5. Out of 23 serotypes, 9 had significant differences (6B, 10A, 12F, 15B, 17F, 18C, 19A, 19F, and, 23F) (Figure 2b; Supplemental Table S1). Serotype 15B had the highest pre-immunization IgG levels, while serotype 3 had the lowest. We performed a more detailed calculation and found that 20.71% of individuals in the middle-aged group (64/309) and 36.36% of individuals in the elderly group (100/275) had a baseline IgG concentration ≥ 1.3 μg/mL in more than 70% of the serotypes. Moreover, 1.62% of individuals in the middle-aged group (5/309) and 0.73% of individuals in the elderly group (2/275) had achieved a baseline IgG concentration ≥ 1.3 μg/mL among all of the 23 serotypes. 

### 3.3. Antibody Response to PPSV23

The comparison of immunogenicity between individuals with baseline IgG ≥ 1.3 µg/mL and individuals with baseline IgG < 1.3 µg/mL showed great differences both for the seroconversion rate and GMFR (Table 1), 15 out of 23 showed differences on seroconversion rates, and larger differences were found on GMFRs that 21 out of 23 serotypes showed differences. Among all of the PPSV23 serotypes, the seroconversion rates ranged from 26.25% to 92.06% in individuals with baseline IgG < 1.3 µg/mL, with the GMFRs ranging from 1.44 to 7.61. The seroconversion rates of those with baseline IgG levels no less than 1.3 µg/mL ranged from 68.18% to 95.58%, and their GMFRs varied from 3.77 to 13.50. There was no difference in immunogenicity by serotype 10A and 19A between the two baseline groups after vaccination. Individuals with baseline IgG < 1.3 µg/mL had higher seroconversion rates and GMFRs than those ≥ 1.3 µg/mL, except serotype 10A. Seroconversion in individuals with baseline IgG < 1.3 µg/mL were over 40% higher than those ≥ 1.3 µg/mL for serotype 3. 

### 3.4. Relationship between Baseline IgG Concentration and Seroconversion Rate

**Figure 3:**
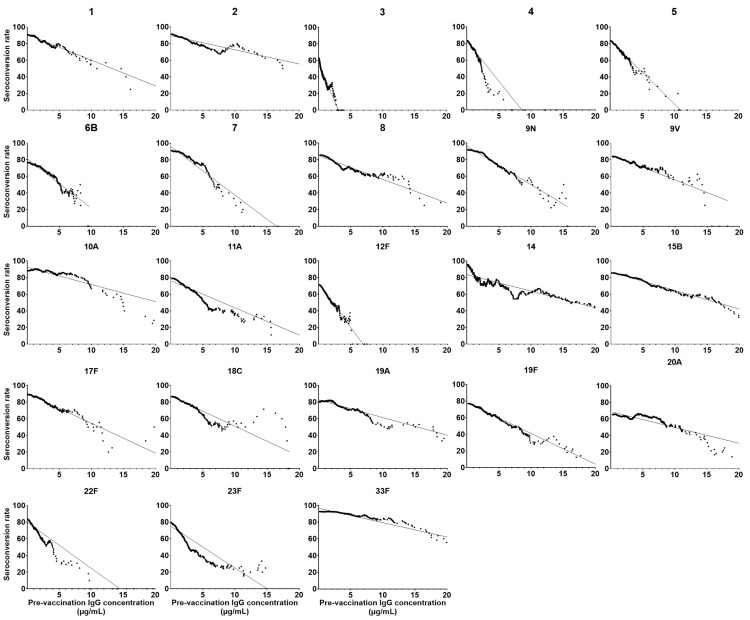
Scatter plots evaluating the relationship between baseline IgG and seroconversion rate in PPSV23-covered serotypes (1, 2, 3, 4, 5, 6B, 7F, 8, 9N, 9V, 10A, 11A, 12F, 14, 15B, 17F, 18C, 19A, 19F, 20A, 22F, 23F, and 33F). Each point represents the seroconversion rate (*y*-axis) of individuals with pre-immunization IgG concentration achieving the value (*x*-axis). The solid line indicates a linear relationship between baseline IgG and seroconversion rate.

Linear regression and correlation coefficients were evaluated to compare the linear relationship between the seroconversion rates and pre-immunization IgG concentrations for all 23 serotypes (Figure 3). The equations and R^2^ values are shown in Table S2. The absolute values of the slope for serotypes 3, 4, and 12F were higher among all 23 types and serotypes 2, 20A, and 33F were lower.

## 4. Discussion

Understanding baseline levels of pneumococcal antibodies has important implications not only for naturally acquired immunity but also for vaccine design and evaluation. Here, we conducted a large-scale cross-sectional study investigating baseline lgG antibody levels to *Streptococcus pneumoniae*. We measured serotype-specific IgG against the 23 vaccine serotypes in 584 adults aged 18 years and older. In more than half of the vaccine serotypes, over 50% of the participants achieved a pre-vaccination IgG concentration of 1.3 μg/mL, which is defined by the AAAAI as a protective concentration from infection. We found that the immunogenicity of individuals with baseline IgG < 1.3 µg/mL and those with baseline IgG ≥ 1.3 µg/mL differed significantly in terms of the seroconversion rate and GMFR. Furthermore, we found when the baseline lgG level increased, the seroconversion rate decreased according the linear regression model. Our results imply that baseline IgG levels have an effect on the evaluation on the assessment of immunogenicity, and therefore a more appropriate criterion needs to be proposed for evaluating the immunogenicity of polysaccharide vaccine.

Vaccine-related anti-Pn IgG concentrations detected by the WHO ELISA reported in preview studies usually focus on a few serotypes. We used an automated workstation to replace the extensive manual operation of the WHO ELISA to quantify anti-Pn IgG concentration levels for all PPSV23-related serotypes. When comparing the results of baseline IgG GMCs with the research conducted by Ciprero et al. in 2016 [16], which had reported five serotypes preimmunization IgG concentrations in Russian subjects over 50 years, we found that the results of serotype 6B and serotype 23F are remarkably comparable. Our results on baseline IgG GMC of serotype 4 and 19F are very similar to those reported by Ridda et al. in 2009, who had described baseline IgG GMC of four serotypes in 241 Australians aged 60 years or older [25]. Comparisons showed that the distribution of baseline pneumococcal IgG antibodies in different countries are partly similar. We have three main findings based on our data regarding the baseline levels of pneumococcal IgG antibodies distribution. First, the concentrations of baseline antibodies against serotype 3 were extremely low. This finding is consistent with that of previous reports [26,27,28]. Low baseline IgG concentration against serotype 3 may result from poor immunogenicity or the fact that serotype 3 is more frequently associated with the invasive disease rather than with the nasopharyngeal carriage. Second, females had generally higher IgG concentrations than males, consistent with Ulanova et al. [26]. This phenomenon seems to match the theory that females generally exhibit greater antibody responses due to higher B cell counts compared to males [29]. Third, the elderly had generally higher IgG concentrations than middle-aged adults. This finding is different from that of previous studies [18,20]. Preview studies based on an experimental human carriage have demonstrated that *S. pneumoniae* colonization increases serum antibody levels [30,31,32]. We speculate that elderly individuals may have experienced more chance on repeated episodes of nasopharyngeal colonization than younger adults, which could lead to increased serum anti-pneumococcal antibody levels. In addition, adults have higher levels of pre-immunization baseline antibodies compared to children under 5-years-old based on the data from our previous study and other studies [27,33]. Given that pneumococcal carriage rates in adults are lower than those in children and the antibody concentrations in adults are higher than those in children, it is suggested that antibodies may facilitate the clearance of the carriage. However, the morbidity and mortality rates of pneumococcal disease are higher among the elderly, despite them having higher baseline antibody levels than adults. Reduced opsonophagocytic activity and the binding strength of the IgG antibody may account for that [34] and according to Simell et al., the concentration of serotype-specific IgG required for the 50% killing of *S.pneumoniae* was significantly higher in the elderly compared to younger adults [35].

Pneumococcal polysaccharide serotype-specific antibodies have been shown to be effective in protecting against IPD. Although the threshold 0.35 µg/mL is used for vaccine efficacy on PCVs in young children, the protective concentration against pneumococcal infection has not been well established in adults [36]. The value of 1.3 μg/mL has been used as a cut-off IgG concentration for protection against infection in a lot of published studies [12,14,19,31]. Data reported by Li et al. on Chinese IPD information showed that the five most prevalent serotypes in adults were 3 (21.7%), 19F (11.8%), 19A (10.5%), 23F (7.2%), and 14 (5.9%) [37]. We found that serotype 3 had the lowest baseline IgG levels, which coincided with the highest prevalence of serotype 3. However, data from our study indicate that adults have a high level of baseline IgG in serotypes 19F and 14. According to the systematic review about streptococcus pneumoniae serotype distribution in children in mainland of China by Shuang et al. [38], serotypes 19F and 14 are the two out of five most common non-invasive strains. This finding lead to a similar conclusion that repeated episodes of nasopharyngeal colonization could lead to increased serum anti-pneumococcal antibody levels. It also suggests that the value of 1.3 μg/mL could not be an effective threshold concentration against carriage. The current criteria for evaluating the immunogenicity of pneumococcal vaccines are mainly related to folds increased from baseline levels. Immunogenicity comparisons between individuals with baseline IgG ≥ 1.3 µg/mL and those with baseline IgG < 1.3 µg/mL showed significant differences in seroconversion rates and GMFR for 15 of the 23 serotypes. Data from our study show that many adults, especially the elderly, have high levels of pre-vaccine antibodies, which have a great effect on the seroconversion rate and GMFR. According to research by Nathaniel et al., there appear to be serotype-specific pre-vaccination concentration thresholds ranging from 4.4 to 10.3 μg/mL, above which a 4-fold or greater response does not occur [13]. Hoffman et al. demonstrated that choosing different criteria can lead to markedly different outcomes of immunogenicity evaluation [12]. Thus, the seroconversion rate alone may not be accurate enough to reflect the immunogenicity of a vaccine. It is crucial to add a threshold concentration of pre-immune antibody levels when evaluating immunogenicity, and above this threshold, using a two-fold increase from baseline to define seroconversion would not be appropriate.

Our study was based on a well-designed randomized controlled trial with big sample sizes, which can generate high-quality data. In our study, IgG levels were quantitatively measured using the WHO ELISA, which has rarely been used in previous studies because of high labor intensity, and high requirements for relevant reagents. Results of previous research using non-uniform techniques such as multiplex bead array methods, commercialization kits, and a multiplex electrochemiluminescence assay, show significant variability, as well as inconsistent correlation with the WHO ELISA [11,39,40]. However, there are some limitations to this study. We have only quantified the total IgG antibodies by ELISA without conducting opsonophagocytic killing assays for testing functional antibodies, which is more indicative of the vaccine’s effectiveness. In future work, we will evaluate the function of baseline IgG antibodies, and further compare naturally acquired antibodies and vaccine-induced antibodies in the efficiency against pneumococcal disease. We used pre-vaccination antibodies to represent baseline levels without excluding participants with a history of pneumococcal vaccination before five years despite the extremely low rate of vaccination for PPSV23 in Beijing before 2016.

## 5. Conclusions

In conclusion, most adults have already achieved protective levels of antibodies to some of the vaccine-covered serotypes before receiving the PPSV23 vaccine, which presents a great challenge to the existing criteria for evaluating the immunogenicity of vaccines and implies that a set of more suitable criteria for evaluating the immunogenicity of pneumococcal vaccines for adults should be developed as soon as possible. However, this finding does not mean that pneumococcal vaccination is no longer necessary for adults, as the proportion of individuals acquiring protective antibody levels for all serotypes covered by PPSV23 before immunization is very low.

## Figures and Tables

**Table 1:** Comparison of seroconversion rates or GMFRs in individuals with baseline IgG ≥ 1.3 µg/mL versus individuals with baseline IgG < 1.3 µg/mL.

Serotype	Pre-Vaccination IgG < 1.3 µg/mL			Pre-Vaccination IgG ≥ 1.3 µg/mL			*p* Value	
	Number	Seroconversion Rate	GMFR	Number	Seroconversion Rate	GMFR	P1 ^a^	P2 ^b^
1	317	92.74	11.09	210	88.10	6.59	NS	<0.05
2	271	95.57	13.18	313	87.86	7.30	<0.05	<0.05
3	484	68.18	3.77	80	26.25	1.44	<0.05	<0.05
4	435	85.52	7.09	82	71.95	3.58	<0.05	<0.05
5	412	87.38	7.39	172	73.84	4.26	<0.05	<0.05
6B	212	79.72	5.17	351	72.36	3.72	NS	<0.05
7	280	92.86	11.44	283	86.93	6.21	<0.05	<0.05
8	118	94.07	10.70	439	82.69	5.15	<0.05	<0.05
9N	226	95.58	12.44	358	89.39	7.02	<0.05	<0.05
9V	193	87.56	5.91	383	81.46	4.23	NS	<0.05
10A	195	84.62	7.24	337	90.21	7.61	NS	NS
11A	168	88.69	6.20	385	74.55	3.58	<0.05	<0.05
12F	391	78.01	4.48	186	58.06	2.49	<0.05	<0.05
14	152	91.45	11.19	416	70.19	4.44	<0.05	<0.05
15B	85	95.29	9.35	484	83.88	5.11	<0.05	<0.05
17F	283	90.81	9.02	263	86.69	5.40	NS	<0.05
18C	213	92.49	8.34	334	82.63	4.43	<0.05	<0.05
19A	274	79.56	6.20	294	81.29	5.60	NS	NS
19F	128	88.28	4.96	433	72.98	3.62	<0.05	<0.05
20A	82	74.39	3.87	478	63.60	2.78	NS	<0.05
22F	334	87.72	7.87	135	71.11	4.35	<0.05	<0.05
23F	358	85.75	6.84	209	67.94	3.17	<0.05	<0.05
33F	93	93.55	13.50	466	92.06	7.42	NS	<0.05

a: Seroconversion rates of pre-vaccination IgG < 1.3 µg/mL vs. ≥ 1.3 µg/mL; b: GMFRs of pre-vaccination IgG < 1.3 µg/mL vs. ≥ 1.3 µg/mL.

## Data Availability

The data that support the findings of this study will be available upon request from the corresponding author.

## Supplementary Materials

The following supporting information can be downloaded at: https://www.mdpi.com/article/10.3390/vaccines11121780/s1, Table S1. The GMCs and 95% CIs for baseline anti-Pn IgG concentrations (μg/mL) in adults over 18 years. Table S2. The equations and R2 of the linear regression model evaluating the relationship between baseline IgG titer andseroconversion rate.
