# Supplementary material for: Baseline Pneumococcal IgG Levels and Response to 23-Valent Pneumococcal Polysaccharide Vaccine among Adults from Beijing, China
Source: Vaccines (Basel). 2023 Nov 29;11(12):1780. doi: 10.3390/vaccines11121780 (PMC10748153; doi:10.3390/vaccines11121780)
Supplement: Supplementary file 1 [file vaccines-11-01780-s001.zip › vaccines-2696950-supplementary.pdf]

Supplemental materials

Baseline Pneumococcal IgG Levels and Response to 23-Valent Pneumococcal Polysaccharide Vaccine among Adults from Beijing, China

Shanshan Zhou [1], Min Lv [1], Shuang Bai [1], Weixin Chen [1], Wei Zhao [1], Jian Wang [1], Ao Zhang [1], Jing Li [1], Hui Xie [1], Yanqing Gao [2], Dongmei Li [2] and Jiang Wu [1,*]

[1] Beijing Center for Disease Prevention and Control, No. 16, Hepingli Middle Street, Dongcheng District, Beijing 100013, China
[2] Daxing District Center for Disease Control and Prevention of Beijing, Beijing 102600, China
* Correspondence: wj81732@hotmail.com

Supplementary Table S1 The GMCs and 95% CIs for baseline anti-Pn IgG concentrations (μg/mL) in adults over 18 years

Serotype	Adult group			Elderly group			Male group			Female group			P value	
	No	Pre-GMC	95% CI	No	Pre-GMC	95% CI	No	Pre-GMC	95% CI	No	Pre-GMC	95% CI	Adult vs Elderly	Male vs Female
1	306	1.03	0.91-1.16	241	1.07	0.94-1.2	306	1.04	0.93-1.17	241	1.06	0.92-1.19	NS	NS
2	309	1.51	1.33-1.72	275	1.48	1.29-1.7	328	1.52	1.33-1.7	256	1.55	1.29-1.72	NS	NS
3	289	0.37	0.33-0.42	275	0.42	0.37-0.48	317	0.46	0.33-0.42	247	0.47	0.37-0.48	<0.05	NS
4	309	0.48	0.43-0.52	232	0.55	0.49-0.63	301	0.52	0.43-0.53	240	0.55	0.48-0.6	NS	NS
5	309	0.81	0.73-0.9	275	0.77	0.69-0.86	328	0.82	0.7-0.86	256	0.81	0.72-0.89	NS	NS
6B	304	1.61	1.46-1.78	275	1.82	1.64-2.02	325	1.60	1.44-1.75	254	1.87	1.68-2.08	NS	<0.05
7	309	1.06	0.94-1.19	254	1.57	1.38-1.78	317	1.22	1.04-1.31	246	1.40	1.23-1.61	<0.001	NS
8	305	2.47	2.23-2.73	269	2.61	2.38-2.87	322	2.49	2.26-2.74	252	2.59	2.35-2.86	NS	NS
9N	301	1.73	1.58-1.9	275	2.19	1.97-2.43	328	1.71	1.54-1.91	256	1.75	1.52-1.95	<0.001	NS
9V	309	1.35	1.21-1.51	275	2.25	2.01-2.52	323	1.85	1.69-2.03	253	2.05	1.85-2.28	<0.01	NS
10A	302	1.71	1.54-1.9	246	2.05	1.82-2.31	311	1.71	1.54-1.9	237	2.05	1.82-2.32	<0.05	<0.05
11A	309	2.06	1.84-2.29	268	2.15	1.91-2.41	323	2.02	1.81-2.26	254	2.20	1.96-2.46	NS	NS
12F	309	0.76	0.69-0.83	275	1.09	0.99-1.2	328	0.83	0.75-0.89	256	1.03	0.9-1.12	<0.001	<0.01
14	304	2.39	2.03-2.82	274	3.33	2.88-3.84	323	2.63	2.22-2.96	255	3.23	2.63-3.72	<0.01	NS
15B	302	3.19	2.86-3.57	275	3.95	3.5-4.46	325	3.21	2.89-3.58	252	4.00	3.53-4.53	<0.01	<0.05
17F	295	1.16	1.03-1.32	268	1.41	1.25-1.58	317	1.20	1.05-1.3	246	1.43	1.25-1.63	<0.05	<0.05
18C	296	1.53	1.38-1.71	275	1.78	1.6-1.98	322	1.52	1.35-1.64	249	1.87	1.67-2.11	NS	<0.01
19A	309	1.23	1.08-1.39	268	1.50	1.31-1.73	326	1.24	1.09-1.39	251	1.53	1.3-1.76	<0.05	<0.05
19F	295	2.23	2.02-2.46	275	2.51	2.25-2.79	320	2.14	1.96-2.35	250	2.66	2.37-3	NS	<0.01
20A	309	2.97	2.69-3.27	266	3.32	2.98-3.69	323	2.99	2.72-3.29	252	3.30	2.96-3.68	NS	NS
22F	280	0.70	0.62-0.8	225	0.96	0.85-1.1	279	0.83	0.69-0.9	226	0.86	0.73-0.96	<0.001	NS
23F	308	0.89	0.78-1	273	1.20	1.05-1.37	326	0.99	0.82-1.05	255	1.16	1-1.31	<0.01	<0.05
33F	301	2.69	2.43-2.97	275	3.59	3.22-4	325	2.89	2.6-3.2	251	3.36	3.02-3.74	<0.001	NS

Supplementary Table S2 The equations and R2 of the linear regression model evaluating the relationship between baseline IgG titer andseroconversion rate

Serotype	equation	R2	P value
1	Y = -3.105*X + 91.20	0.9508	<0.05
2	Y = -1.705*X + 89.55	0.9093	<0.05
3	Y = -18.98*X + 58.37	0.9187	<0.05
4	Y = -9.679*X + 82.95	0.8556	<0.05
5	Y = -7.645*X + 83.90	0.9682	<0.05
6B	Y = -5.964*X + 81.12	0.9234	<0.05
7	Y = -5.827*X + 95.82	0.9156	<0.05
8	Y = -2.850*X + 84.78	0.9305	<0.05
9N	Y = -4.628*X + 96.13	0.9483	<0.05
9V	Y = -3.040*X + 85.98	0.8637	<0.05
10A	Y = -2.063*X + 92.22	0.8477	<0.05
11A	Y = -3.273*X + 76.31	0.826	<0.05
12F	Y = -10.57*X + 72.94	0.9751	<0.05
14	Y = -1.998*X + 83.71	0.8429	<0.05
15B	Y = -2.226*X + 86.70	0.9586	<0.05
17F	Y = -3.599*X + 90.57	0.94	<0.05
18C	Y = -3.613*X + 87.00	0.8276	<0.05
19A	Y = -2.123*X + 82.57	0.9135	<0.05
19F	Y = -3.699*X + 78.11	0.9726	<0.05
20A	Y = -1.905*X + 68.76	0.7877	<0.05
22F	Y = -5.504*X + 79.69	0.8562	<0.05
23F	Y = -5.002*X + 75.07	0.8142	<0.05
33F	Y = -1.726*X + 96.59	0.9324	<0.05
